# Molecular detection and characterization of *Theileria annulata, Babesia bovis,* and *Babesia bigemina* infecting cattle and buffalo in southern Egypt

**DOI:** 10.1016/j.parepi.2024.e00340

**Published:** 2024-02-01

**Authors:** Hassan Y.A.H. Mahmoud, Abdelrahman A. Rady, Tetsuya Tanaka

**Affiliations:** a Division of Infectious Diseases, Animal Medicine Department, Faculty of Veterinary Medicine, South Valley University, Qena 83523, Egypt; b Laboratory of Infectious Diseases, Joint Faculty of Veterinary Medicine, Kagoshima University, Kagoshima 890-0065, Japan

**Keywords:** *Babesia bovis*, *Babesia bigemina*, *Theileria annulata*, Egypt, PCR

## Abstract

Tick-borne diseases have a major adverse effect on livestock worldwide, causing enormous economic losses in meat and milk production as well threatening animal and public health. In this study, we aimed to detect and characterize piroplasms isolated from cattle and buffalo in southern Egypt, using molecular techniques. Three hundred blood samples were collected from cattle and buffalo in two governorates in southern Egypt. All 300 samples (100%) were confirmed to contain DNA, as they exhibited bands of bovine *β-actin* gene at the expected 227 bp for cattle and buffalo. The samples were analyzed by PCR for the presence of piroplasms, specifically *Babesia bovis*, *Babesia bigemina*, and *Theileria annulata*. Samples positive for the *piroplasma 18S ribosomal RNA* gene were further examined for two additional genes, *spherical body protein 4* gene, to provide an enhanced degree of specificity for the identification of *B. bovis* and *B. bigemina*, and the *major merozoite surface antigen* gene for *T. annulata.* The infection rate for piroplasma spp. was 60/300 (20%). The positivity rates were 10.7% (32/300) for *T. annulata*, 5.3% (16/300) for *B. bovis*, and 4% (12/300) for *B. bigemina*. By host species, 42/150 (28%) cattle and 18/150 (12%) buffalo were positive for piroplasms. None of the isolates sequenced for the *B. bovis* isolates from buffalo in this study showed 100% identity with any sequence deposited in GenBank for the *small subunit ribosomal RNA* gene (maximum identity value = 99.74%). Similarly, no *T. annulata small subunit ribosomal RNA* gene sequence identified in this study exhibited 100% identity with any sequence deposited in GenBank (maximum identity value = 99.89%). The current study provides a partial sequence of the *T. annulata merozoite-piroplasm surface antigen* gene, as well as the *B. bovis* and *B. bigemina spherical body protein 4* genes, in cattle and buffalo in southern Egypt, and is the first report on these piroplasma genes in cattle and buffalo in southern Egypt.

## 1 Introduction

*Theileria* parasites are hemoparasites that can survive transcrardially in ticks and can cause the potentially fatal disease of theileriosis when spread to humans, or domestic or wild animals through tick blood feeding (Nayel et al., 2012). One of these parasites, *T. annulata*, causes tropical theileriosis, which affects North Africa, mainly Egypt (Gharbi et al., 2013; Ayadi et al., 2016). Cattle that have been chronically infected and recovered may become long-term carriers of *T. annulata* (Tavassoli et al., 2011); these asymptomatic carriers are heavily implicated in the transmission of theileriosis among large ruminants and serve as reservoirs for re-infection of feeding ticks (Gul et al., 2015).

Theileriosis results in significant financial losses for farmers due to the debilitation, sudden mortality, morbidity, and milk in livestock, and the need for expenditure on acaricide, therapy, and vaccines (Ayadi et al., 2016). Only a few studies have reported on the prevalence of theileriosis in buffalo with one such report stemming from India (Durrani et al., 2008) and another from Egypt (Mahmmod et al., 2011); furthermore, little is known about the diagnosis and epidemiology of the disease in water buffalo.

Buffalo are natural hosts of *T. annulata* but remain frequent carriers due to their ability to control the infection through a cellular- and humeral-mediated immune response (Stagg et al., 1983). Clinically, affected animals display depression, lacrimation, diarrhea, anorexia, and weight loss; however, the most prevalent signs are abortion, corneal opacity, severe pulmonary edema with dyspnea, and foamy nasal discharge. Enlargement of the superficial lymph nodes has also been reported to accompany these symptoms. Icterus, anemia, and rarely hemoglobinuria are also symptoms of theileriosis (Maxie, 2015).

The major merozoite/piroplasm surface protein (30–32 kDa) of *T. annulata* is immunogenic and antigenically diverse (Dickson and Shiels, 1993). Allelic variants are responsible for the size polymorphism it can exhibit. The protein plays a role in host cell invasion by recognizing and interacting with the merozoite surface and the membrane proteins of red blood cells (Shiels et al., 1995). In several investigations, *the major merozoite surface* gene was discovered to be highly variable, which raises concerns about the applicability of *major merozoite surface antigen* gene-targeted primers for detecting all *T. annulata* isolates (Santos et al., 2013). Despite tropical theileriosis's widespread distribution and endemicity, little is known about variation in the *major merozoite surface* gene of *T. annulata* (Gubbels et al., 2000).

Babesiosis is endemic to tropical and subtropical regions worldwide (Fakhar et al., 2012), and its most significant causative (*Babesia*) pathogens in cattle are reported to be *B. bigemina* and *B. bovis* (Fakhar et al., 2012). They have been reported in southern Europe, Australia, Asia, Africa, Central and South America, and Australia (Uilenberg, 1995). Babesiosis is now regarded as the most worrying endemic parasitic disease affecting cattle in Egypt because of its enormous economic impact on meat and milk production as well as livestock management (Adham et al., 2009).

*B. bigemina* and *B. bovis* can produce acute diseases that can lead to severe clinical symptoms, such as potentially fatal hemolytic anemia (Góes et al., 2007). Acute cases frequently develop into chronic ones, especially in bovines below the age of one year, and these animals continue to harbor the parasites, thus entailing the risk of zoonoses as the parasites are circulated by tick transmission. According to earlier research, many cattle in Egypt may have subclinical babesiosis (Adham et al., 2009). Furthermore, despite clinical evidence suggesting that buffalo are more resistant to *Babesia* infections (Mahmmod, 2013), there is a lack of fully confirmatory data on the hemoparasites in this animal.

*B. bovis* infection is more severe than *B. bigemina* infection because erythrocytes infected by the former are sequestered in the kidney, lung, and brain micro-capillaries, leading to organ failure and fatal systemic shock. Most cattle recovering from *B. bovis* infection continue spreading the parasite and act as reservoirs for its spread to other animals (Bock et al., 2004). *Babesia* parasites are recognized by the rhoptries, micronemes, and spherical body organelles that comprise the apical complex, which serve as their defining characteristic structure. In addition to the parasites' membrane component, proteins created by these organelles play essential roles in parasites' survival and development (Yokoyama et al., 2006). These proteins include spherical body proteins that facilitate the growth of parasites and regulate the environment during invasion (Goo et al., 2008; Terkawi et al., 2009). They are also regarded as promising candidates for creating diagnostic antigens or subunit vaccinations (Yokoyama et al., 2006).

In this study, we used molecular epidemiology techniques to further elucidate the genetics of *Theileria* and *Babesia* spp. and other hemoparasites circulating in southern Egypt, which have recently become resistant to treatment. We focused on infections in cattle and buffalos, in light of their economic importance, and the risks posed when these animals are imported into Egypt and then come into contact with local breeds, as is often the case when buffaloes are maintained in close proximity to cattle in the country's farming sector.

## 2 Materials and methods

### 2.1 Study design and research area

**Fig. 1:**
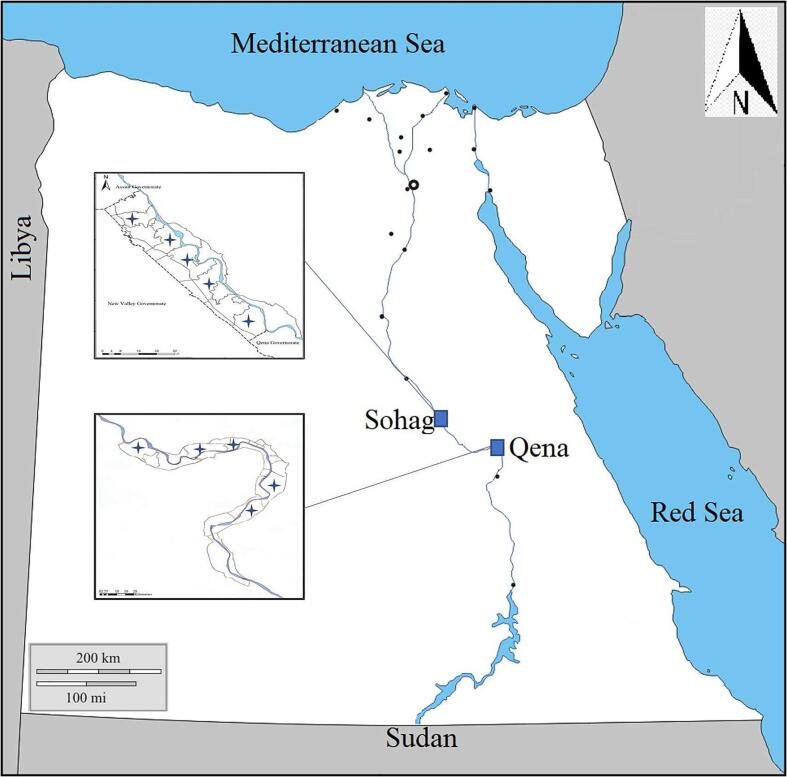
Map of Egypt showing the southern part of Egypt where the blood samples were collected from animals in two governorates, Sohag and Qena.

In the current study, we concentrated on piroplasmosis infection in local breeds of cattle and buffalo, in the southern Egypt governorates of Sohag and Qena between April 2021 and January 2022 (Fig. 1). We targeted animals of both sexes and at different ages (from one to three years) for investigation.

### 2.2 Clinical examination

Before blood samples were taken, animals underwent clinical examinations. Examinations included determining age and sex, measuring body mass index, body temperature, heart rate, and respiratory rate, and checking visible mucous membranes.

### 2.3 Collection of samples

Animals chosen at random were targeted for collection of samples. Each sample was drawn as whole blood from the jugular vein using a sterile, clean vacutainer tube that contained anticoagulants for DNA extraction and PCR amplification. All samples were stored at −30 °C from collection until usage.

### 2.4 Detection of control genes and pathogens by PCR

All primers used in this study are listed in Table 1, and the PCR conditions are shown in Table 2. The amplification of bovine *β-actin* for DNA extracts was confirmed by amplifying the bovine (housekeeping) genes to ensure that the genomic DNA had been extracted from all samples (Squillacioti et al., 2014). Negative controls were preparations containing nuclease-free water. PCR products was subjected to electrophoresis with a 1.5% agarose gel in 1 × Tris–acetate–EDTA (TAE) buffer using a Mupid electrophoresis device (Mupid Co., Ltd., Tokyo, Japan), and bands were visualized through a gel documentation system UV device, WUV-M20 (ATTO Co., Ltd., Tokyo, Japan), after being stained with 5 mg/ml ethidium bromide in 1 × TAE.

**Table 1:** Primers for the detection of *Piroplasma* in cattle and buffalo.

Organism	Target gene	Primer name	Sequence (5′ → 3′)	Expected size (bp)	References
Blood of cattle and buffalo	*Bovine β-actin gene*	FBA	CGCACCACCGGCATCGTGAT	227	Squillacioti et al., 2014
		RBA	TCCAGGGCCACGTAGCAGAG		
Piroplasma (*Babesia/ Theileria/Hepatozoon*)	*18S ribosomal RNA*	Piro18S Fa Forward	GGTGAAACTGCGAATGGCTC	1455–1680	Galay et al., 2018
		Piro18S Ra Reverse	AAGTGATAAGGTTCACAAAACTT		
		Pro18SFb Forward	TGGCTCATTACAACAGTTATA		
		Piro18SRb Reverse	CGGTCCGAATAATTCACC		
*Babesia bovis*	*Spherical body protein 4*	SBP4- Forward 1	AGTTGTTGGAGGAGGCTAAT	503	Terkawi et al., 2011
		SBP4- Reverse 1	TCCTTCTCGGCGTCCTTTTC		
		SBP4- Forward 2	GAGTCTGCCAAATCCTTAC		
		SBP4- Reverse 2	TCCTCTACAGCTGCTTCG		
*Babesia bigemina*	*Spherical body protein 4*	SBP4- F 1	GTGCTGCTTAATCGCACAAAC	853	Mtshali and Mtshali, 2013
		SBP4- R 2	AAGATGCCTTCTTCGGTGATG		
		SBP4- F 2	CGGATCCTGTTATCGTTCCTG		
		SBP4- R 2	GAAGTTACGCCTGGAGTTGG		
*Theileria annulata*	*Major merozoite surface antigen—Tams1*	Tams1 F	GTAACCTTTAAAAACGT	721	d'Oliveira et al., 1995
		Tams1 R	GTTACGAACATGGGTTT

**Table 2:** PCR conditions for the amplification of target fragment genes of *Piroplasma* in cattle and buffalo.

Target gene	PCR condition
*Bovine β-actin*	$\frac{94^{{}^{\circ}}C}{5\;\min}\to \left[\;\frac{94^{{}^{\circ}}C}{30\;s}-\frac{65^{{}^{\circ}}C}{30\;s}-\frac{72^{{}^{\circ}}C}{30s}\;\right]\;35\times \to \frac{72^{{}^{\circ}}C}{5\;\min}\to 10^{{}^{\circ}}C\;$∞
*18S ribosomal RNA*	$1\mathrm{st}\;\text{round}:\frac{94^{{}^{\circ}}C}{\;5\;\min}\to \left[\;\frac{98^{{}^{\circ}}C}{10\;s}-\frac{55^{{}^{\circ}}C}{15\;s}-\frac{68^{{}^{\circ}}C}{45\;S}\;\right]\;30\times \to \frac{68^{{}^{\circ}}C}{5\;\min}\to 10^{{}^{\circ}}C\;$∞
	$2\mathrm{nd}\;\text{round}:\frac{94^{{}^{\circ}}C}{5\;\min}\to \left[\;\frac{98^{{}^{\circ}}C}{10\;s}-\frac{55^{{}^{\circ}}C}{15\;s}-\frac{68^{{}^{\circ}}C}{45\;S}\;\right]\;30\times \to \frac{68^{{}^{\circ}}C}{5\;\min}\to 10^{{}^{\circ}}C\;$∞
*B. bovis* (*spherical body protein 4*)	$\frac{95^{{}^{\circ}}C}{5\;\min}\to \left[\;\frac{94^{{}^{\circ}}C}{1\min}-\frac{55^{{}^{\circ}}C}{1\min}-\frac{72^{{}^{\circ}}C}{1\min}\;\right]\;35\times \to \frac{72^{{}^{\circ}}C}{10\;\min}\to 10^{{}^{\circ}}C\;$∞
	$\frac{95^{{}^{\circ}}C}{5\;\min}\to \left[\;\frac{94^{{}^{\circ}}C}{1\min}-\frac{55^{{}^{\circ}}C}{1\min}-\frac{72^{{}^{\circ}}C}{1\min}\;\right]\;35\times \to \frac{72^{{}^{\circ}}C}{10\;\min}\to 10^{{}^{\circ}}C\;$∞
*B. bigemina* (*spherical body protein 4*)	$\frac{95^{{}^{\circ}}C}{3\;\min}\to \left[\;\frac{94^{{}^{\circ}}C}{30\;S}-\frac{55^{{}^{\circ}}C}{30\;S}-\frac{72^{{}^{\circ}}C}{1\min}\;\right]\;40\times \to \frac{72^{{}^{\circ}}C}{5\;\min}\to 10^{{}^{\circ}}C\;$∞
*Major merozoite surface antigen—Tams1*	$\frac{95^{{}^{\circ}}C}{5\;\min}\to \left[\;\frac{94^{{}^{\circ}}C}{1\min}-\frac{55^{{}^{\circ}}C}{1\min}-\frac{72^{{}^{\circ}}C}{1\min}\;\right]\;30\times \to \frac{72^{{}^{\circ}}C}{5\;\min}\to 10^{{}^{\circ}}C\;$∞

### 2.5 DNA extraction and PCR amplification

Three hundred samples (150 cattle and 150 buffalo) were analyzed by PCR for the presence of *piroplasms*, specifically *B. bovis, B. bigemina, and T. annulata*. Samples were collected using commercial extraction kits (Wizard® Genomic DNA Purification Kit, Promega, Madison, WI, USA). DNA was then extracted from whole blood samples. The samples were screened for the presence of a *piroplasm (Babesia, Theileria, and Hepatozoon species)* with nested PCR amplification of the *18S ribosomal RNA* gene using the relevant primers (Galay et al., 2018). Selected *B. bovis– and B. bigemina-*positive samples were also subjected to nested PCR targeting the *spherical body protein-4* genes (Mtshali and Mtshali, 2013). *T. annulate-*positive samples were also subjected to conventional PCR targeting the *major merozoite surface antigen* genes (d'Oliveira et al., 1995). The PCR reaction was performed with a total volume of 10 μl, using Tks Gflex DNA Polymerase (TaKaRa), forward and reverse primers at 10 pmol/primer, and nuclease-free water, and a template (1 μl DNA) was used. The PCR conditions are shown in Table 2. A negative control containing nuclease-free water was added to each PCR reaction. The electrophoresis of the PCR products was performed using 1.5% gel and 1 × TAE buffer. Observations were made using a gel documentation system UV device, WUV-M20 (Atto Co., Ltd.), after the gel was stained with 5 μg/ml ethidium bromide in 1 × TAE.

### 2.6 Sequence and data analysis

We submitted 50-μl mixtures prepared from the samples for PCR targeting the *spherical body protein-4* and *major merozoite surface antigen* genes of *B. bovis, B. bigemina, and T. annulata*, or for sequence analysis. The amplicons were purified using a NucleoSpin Gel and PCR Clean-up kit (Macherey-Nagel, Leicestershire, Duren, Germany), following the manufacturer's protocol. Sequence readings were compared with those of reported isolates from a gene bank. A maximum-likelihood phylogenetic tree was constructed using MEGAX software (Kumar et al., 2018), with bootstrap values estimated using 1000 replicates based on Kimura's two-parameter substitution model (Kimura, 1980).

## 3 Results

### 3.1 DNA confirmation and identification

All 300 samples (100%) were confirmed to contain DNA, as they exhibited bands at the expected 227 bp for bovine *β-actin* in cattle and buffalo.

The samples were then subjected to nested PCR to detect the presence of the *piroplasma 18S ribosomal RNA* gene, and 60/300 samples (20%) were found to positive for piroplasma infection overall, with 42/150 (28%) positive samples from cattle and 18/150 (12%) from buffalo. The overall positivity rates for specific hemoparasites were 10.7% (32/300) for *T. annulata*, 5.3% (16/300) for *B. bovis*, and 4% (12/300) for *B. bigemina* (Table 3).

**Table 3:** Detection of piroplasm infections in cattle and buffalo from southern Egypt based on PCR detection in blood samples.

Species	Number of animals	Number negative	*Theileria annulata* number positive	*Theileria annulata* percent positive	*Babesia bovis* number positive	*Babesia bovis* percent positive	*Babesia bigemina* number positive	*Babesia bigemina* percent positive	Total number positive	Total percent positive
Cattle	150	108	24	16%	10	6.7%	8	5.3%	42	28%
Buffalo	150	132	8	5.3%	6	4%	4	2.7%	18	12%
Total	300	240	32	10.7%	16	5.3%	12	4%	60	20%

Samples that were positive for the *piroplasma 18S ribosomal RNA* gene were further examined for two additional genes: the *spherical body protein 4* gene, to provide an enhanced degree of specificity for the identification of *B. bovis* and *B. bigemina*; and the *major merozoite surface antigen* gene, to provide an enhanced degree of specificity of *T. annulata*.

Furthermore, a higher prevalence of piroplasma infection was found in Sohag than in Qena governorate. We found that females had a higher infection rate than males. Further investigations of risk factors should encompass univariate and multivariate analyses at the animal and farm levels. Since we found a high infection rate in older animals (more than one year old) relative to young animals, the husbandry regime also appears to be associated with the risk of piroplasma infection. Individually maintained animals had a lower infection rate than intensively maintained animals (14.7% vs. 25.3%; Table 4).

**Table 4:** Risk factor in piroplasm infections in cattle and buffalo in southern Egypt.

Factors	Locations				Age						Gender				Breeding system			
	Sohag		Qena		1 year		2 years		3 years		Male		Female		Individual		Intensive	
	N	%	N	%	N	%	N	%	N	%	N	%	N	%	N	%	N	%
Number of animals testing positive	32	21.4	28	18.7	8	16	30	20	22	22	11	19.3	49	20.2	22	14.7	38	25.3
Number of animals testing negative	118	78.6	122	81.3	42	84	120	80	78	78	46	80.7	194	79.8	128	85.3	112	74.7
Total number of animals tested	150	100	150	100	50	100	150	100	100	100	57	100	243	100	150	100	150	100

N = Number, % = Percent.

### 3.2 Sequence analysis

**Fig. 2:**
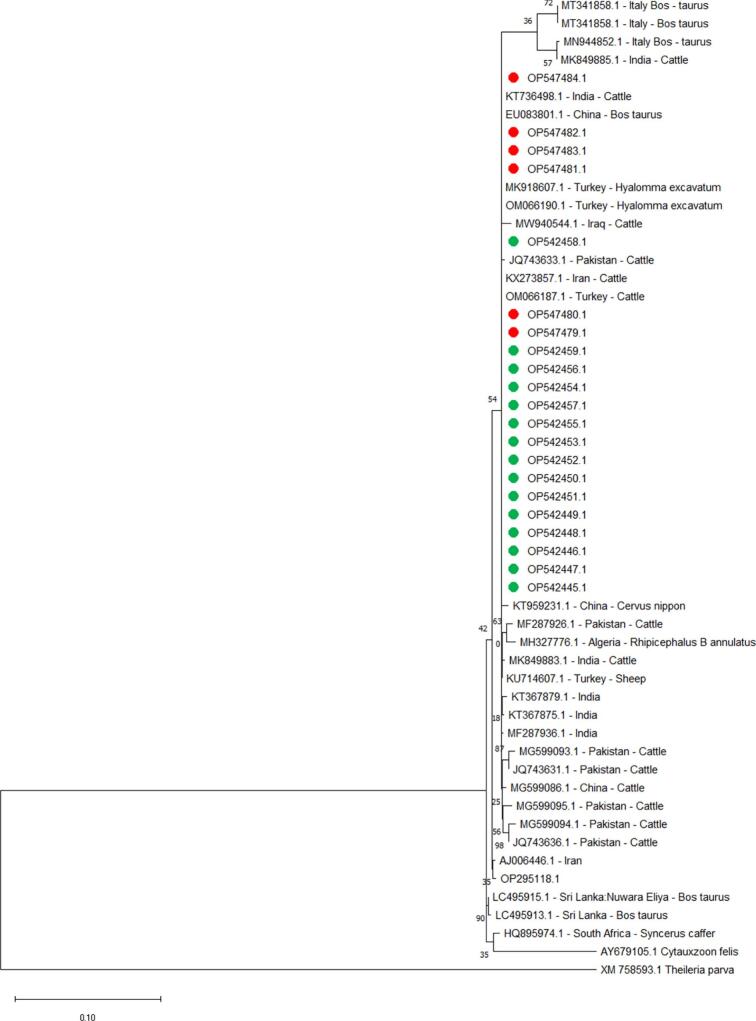
Phylogenetic relationships of *T. annulata* using the maximum likelihood method and the Kimura 2-parameter model based on *small subunit ribosomal RNA*. The percentage of trees in which the associated taxa clustered is shown next to the branches. The tree is drawn to scale, with branch lengths measured in the number of substitutions per site. *T. annulata* obtained in the present study were represented by green circles for cattle and red circles for buffalo. (For interpretation of the references to colour in this figure legend, the reader is referred to the web version of this article.)

**Fig. 3:**
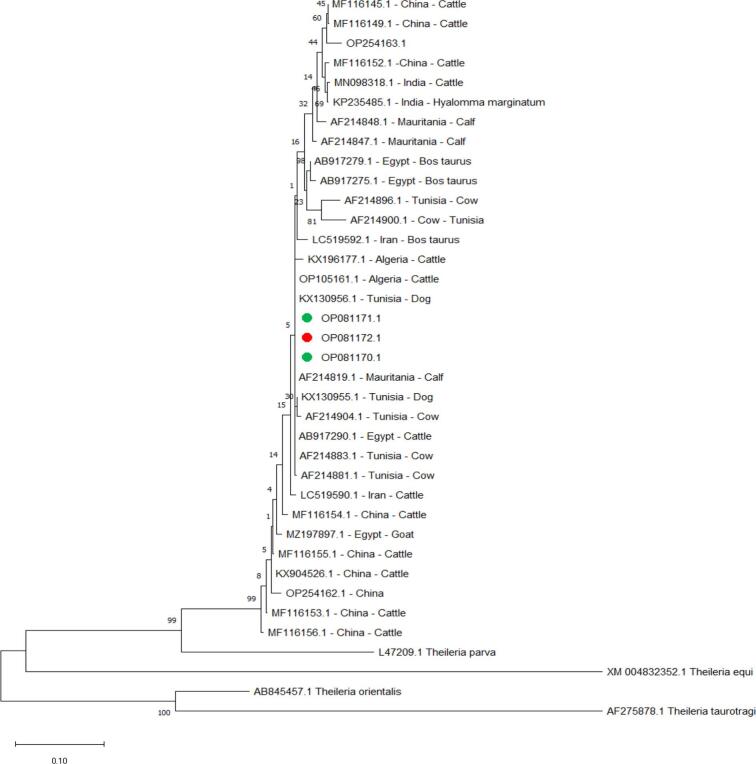
Phylogenetic relationships of *T. annulata* using the maximum likelihood method and the Kimura 2-parameter model based on the *major merozoite-Piroplasm surface antigen*. The percentage of trees in which the associated taxa clustered together is shown next to the branches. The tree is drawn to scale, with branch lengths measured in the number of substitutions per site. *T. annulata* obtained in the present study are represented by green circles for cattle and red circles for buffalo. (For interpretation of the references to colour in this figure legend, the reader is referred to the web version of this article.)

Sequence analysis for the *T. annulata 18S ribosomal RNA* gene (Accession Number OP081171.1, OP081172.1 and OP081173.1) revealed the identity values are ranged from 99.89% to other sequencing in genes submitted to GenBank (Accession Number MT341858.1 and MN944852.1) to 99.36% with (Accession Number MF287926.1) (Fig. 2). The *T. annulata Tams1* gene in this study was 100.00% identical to Algeria cattle (Accession Number KX130956.1), Tunisia dog (Accession Number KX130956.1), and Mauritania calf (Accession Number AF214819.1) and the minimum identical was 95.74% with (Accession Number OP254163.1) (Fig. 3).

**Fig. 4:**
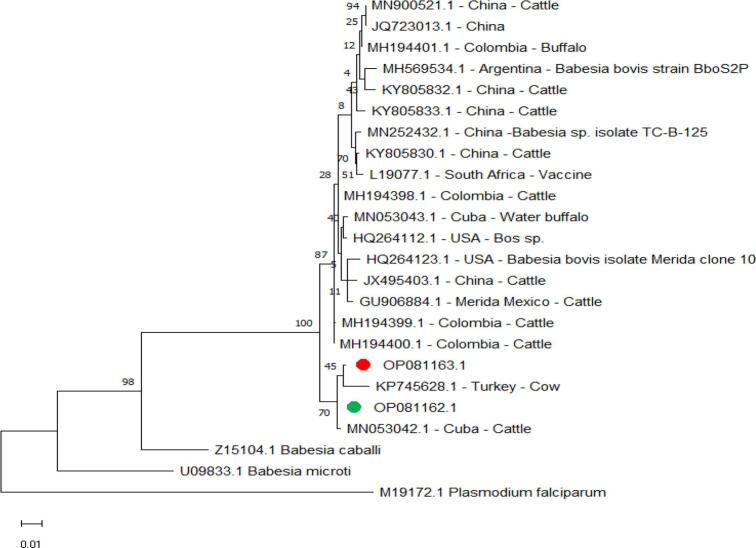
Phylogenetic relationships of *B. bovis* using the maximum likelihood method and the Kimura 2-parameter model based on *small subunit ribosomal RNA*. The percentage of trees in which the associated taxa clustered is shown next to the branches. The tree is drawn to scale, with branch lengths measured in the number of substitutions per site. *B. bovis* obtained in the present study are represented by green circles for cattle and red circles for buffalo. (For interpretation of the references to colour in this figure legend, the reader is referred to the web version of this article.)

The *B. bovis* s*mall subunit ribosomal RNA* gene sequences identified in isolates from buffalo (Accession Number OP081163.1) in this study did not show 100% identity with any sequence previously deposited in GenBank. The maximum identity value was 99.74%, with a sequence reported in Cuban cattle (Accession Number MN053042.1), 99.87% with cattle from this study (Accession Number OP081162.1), and 99.49% with cattle from Turkey (Accession Number KP745628.1) and the minimum identity (98.97%) occurred with cattle from China (Accession Number KY805832.1) (Fig. 4).

**Fig. 5:**
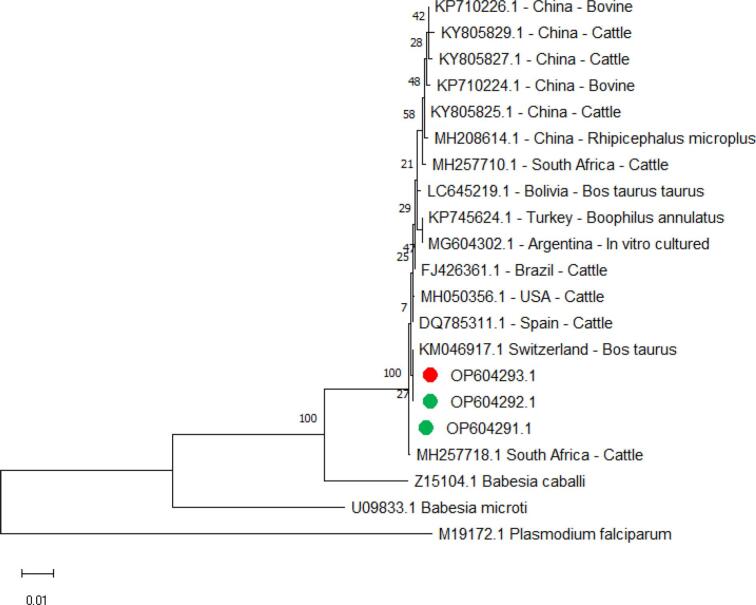
Phylogenetic relationships of *B. bige*min*a* using the maximum likelihood method and the Kimura 2-parameter model based on *small subunit ribosomal RNA*. The percentage of trees in which the associated taxa clustered is shown next to the branches. The tree is drawn to scale, with branch lengths measured in the number of substitutions per site. *B. bige*min*a* obtained in the present study are represented by green circles for cattle and red circles for buffalo. (For interpretation of the references to colour in this figure legend, the reader is referred to the web version of this article.)

*B. bigemina* isolates from cattle in this study, with (Accession Number OP604291.1) for the *small subunit ribosomal RNA* gene, showed a partial sequence identity of 100% with a sequence of *B. bigemina* identified in South Africa (Accession Number MH257718.1), and *B. bigemina* isolates from cattle and buffalo in this study (Accession numbers OP604292.1 and OP604293.1) showed 99.89% identity with isolates from cattle in Switzerland (Accession Number KM046917.1). The maximum identity for three isolates in this study with another isolate is 99.78% for cattle from the USA (Accession Number MH050356.1), and the minimum identity was 99.11% for in vitro cultured cattle from Argentina (Accession Number MG604302.1) (Fig. 5).

**Fig. 6:**
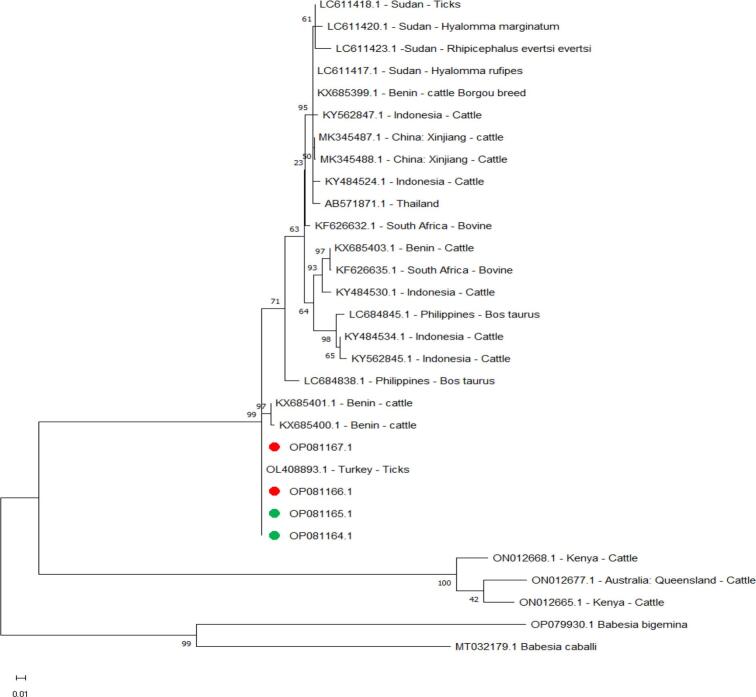
Phylogenetic relationships of *B. bovis* using the maximum likelihood method and the Kimura 2-parameter model based on the *spherical body protein 4* gene. The percentage of trees in which the associated taxa clustered is shown next to the branches. The tree is drawn to scale, with branch lengths measured in the number of substitutions per site. *B. bovis* obtained in the present study are represented by green circles for cattle and red circles for buffalo. (For interpretation of the references to colour in this figure legend, the reader is referred to the web version of this article.)

We identified four *B. bovis spherical body protein 4* genes two for each animals cattle and boffola (Accession Numbers OP081164.1, OP081165.1, OP081166.1, and OP081167.1). Their identity values with previously reported values are 100% identical with Turkey ticks (Accession Number OL408893.1), were maximally and were minimally identical by 90.55% with bovine in South Africa (Accession Number KF626635.1) (Fig. 6).

**Fig. 7:**
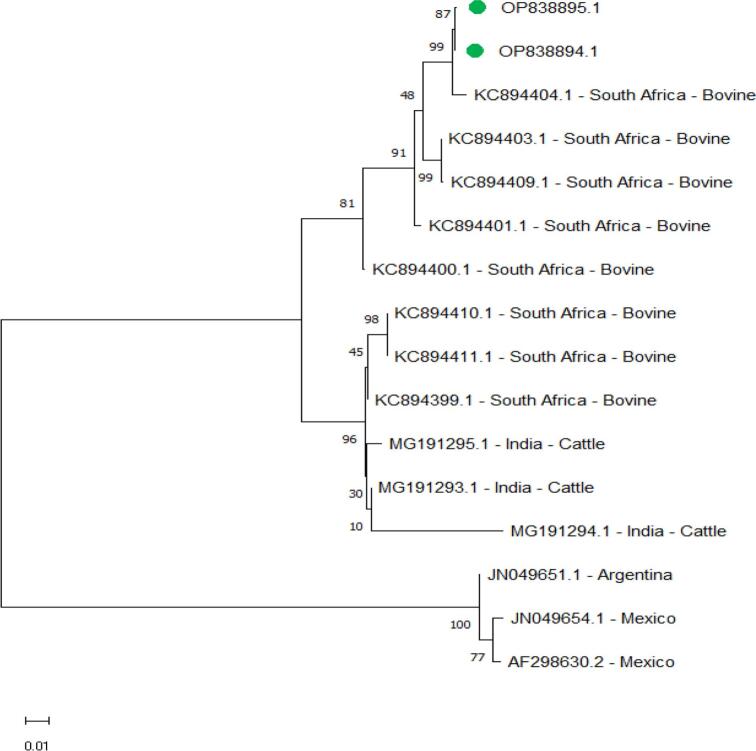
Phylogenetic relationships of *B. bigemina* using the maximum likelihood method and the Kimura 2-parameter model based on the *spherical body protein 4*. The percentage of trees in which the associated taxa clustered is shown next to the branches. The tree is drawn to scale, with branch lengths measured in the number of substitutions per site. *B. bigemina* obtained in the present study are represented by green circles for cattle. (For interpretation of the references to colour in this figure legend, the reader is referred to the web version of this article.)

Two *B. bigemina spherical body protein 4* isolates (Accession Number OP838894.1 and OP838895.1), from cattle sequences in this study were not identical by 100% with any sequences in GenBank, the maximum identity with another isolate in GenBank was 99.23% with bovine isolated from South Africa (Accession Number KC894404.1), and the minimum identity was 87.36% with Indian cattle (Accession Number MG191294.1) (Fig. 7).

## 4 Discussion

In this study, we aimed to investigate the molecular genetics of endemic hemoparasites in Southern Egypt, based on samples from domesticated animal populations (cattle and water buffalos). Specifically, we targeted theilerian and babesian piroplasms and our findings assist the identification of new strains, monitoring of prevalence in regional sentinel populations, and the development of effective control measures, for these parasites.

We evaluated the sequences of the *Tams1* gene of *T. annulata* isolates identified in the present study through comparisons with each other and with other sequences available in the GenBank. Our multiple sequence alignment of the *T. annulata merozoite-piroplasm surface* gene yielded three sequences in samples from cattle and buffalos that we deposited in GenBank (Accession Numbers OP081170.1, OP081171.1, and OP081172.1). Our sequences showed 100% identity to other sequences in GenBank; specifically, those identified in Algerian cattle (Accession Numbers KX130956.1 and OP105161.1), Tunisian dogs (Accession Numbers KX130956.1), and Mauritania calves (Accession Numbers AF214819.1). We also identified sequences of the *T. annulata small subunit ribosomal RNA* gene and deposited them in GenBank (Accession Numbers OP542445.1- OP542459.1 for samples from cattle, and Accession Numbers OP547479.1-OP547484.1 for samples from buffalo). However, none of these sequences showed 100% identity with any previously deposited sequence in GenBank; the maximum identity value they showed was 99.89%.

In molecular epidemiological studies of *T. annulata*, the *18S rRNA* gene has generally been targeted for analysis because it is highly conserved across isolates globally (Kundave et al., 2015). Sequencing and phylogenetic analyses targeting the *T. annulata major merozoite surface antigen* gene have also been reported from India (Arun Raj et al., 2018; Kumar et al., 2019; Roy et al., 2019), Egypt (El-Dakhly et al., 2020), Iran (Habibi, 2013), and Tunisia (Sallemi et al., 2017). The *T. annulata* major merozoite surface antigen (Tams1) has also attracted attention for its potential as a candidate diagnostic marker and vaccine candidate (d'Oliveira et al., 1997). However, its genetic diversity could hinder its utility for vaccination and diagnostic procedures. Additionally, the biological and functional activities of the Tams1 protein can be altered by variations in its sequence. No geographic specificity has been identified in previous research, and nearly identical sequences were reportedly found across a range of geographic regions (Gubbels et al., 2000; Sallemi et al., 2017).

The *B. bovis* s*mall subunit ribosomal RNA* gene sequences identified in isolates from buffalo in this study did not show 100% identity with any sequence previously deposited in GenBank, this result will need more future research in is required to produce a more data related to parasitic infection in boffola, the *18S small-subunit ribosomal RNA* genes have allowed for a more precise classification of piroplasms (Yin et al., 2004).

There are both variable and conserved regions in the eukaryotic *18S rRNA gene*. It has been utilized as a universal biomarker to screen closely related species in biodiversity studies because of its excellent specificity and sequence conservation (Schnittger et al., 2022). The first screening of piroplasmids infecting horses and Bactrian camels in northeastern Mongolia was also conducted using universal oligonucleotide primers based on 18S rRNA (Sloboda et al., 2011).

*B. bovis* and *B. bigemina spherical body protein 4* genes in both cattle and boffola were identical by with previous data in GenBank, the high degree of diversity in the membrane proteins of *Babesia* merozoites allows them to evade the host immune system (Suarez et al., 1998). *Babesia* apical complex organelles generate and release proteins that are important in erythrocyte invasion during asexual reproduction in the vertebrate host during different stages of the life cycle (Yokoyama et al., 2006). Since the first *B. bovis* spherical body protein was identified and characterized (Hines et al., 1995), evidence has suggested that these organelle proteins are released and transported in the erythrocyte membrane, and for *B. bovis* at least, four spherical body proteins known as spherical body proteins 1, 2, 3, and 4 are secreted during this process (Rombel et al., 2000).

In this study, we found that cattle had a greater rate of *T. annulata* infection (16% vs. 5.3% for buffalo) and the total infection rate for both cattle and buffalo for *T. annulata* infection rate was 10.7%. According to our records, the infection rates were higher than the reported of 9.56% in Egypt and 9.8% in Sri Lanka (Elsify et al., 2015; Sivakumar et al., 2012) and lower than previously reported in Egypt and India, 74.63% and 63.79%, respectively (Mahmmod et al., 2010; Khatoon et al., 2015; Kundave et al., 2015) and lower than the infection rates have been noted in the following reports: 32.6% in central Tunisia, 44% in India and 45.33% in Iran (Elati et al., 2023; Roy et al., 2000; Nourollahi-Fard et al., 2015).

The 6.7% *B. bovis* infection rate we found in cattle was lower than previously reported of 20% and 25.33% from Egyptian researchers (Adham et al., 2009; El-Fayomy et al., 2013). Additionally, techniques using nested PCR for the diagnosis of *Babesia* species in cattle identified infection rates of 78.5% and 29% in Portugal and Pakistan, respectively (Silva et al., 2009; Chaudhry et al., 2010). Such wide variation in prevalence rates may be expected considering regional diversity and other environmental factors that may affect tick vector population densities (L'Hostis and Seegers, 2002). Rising temperatures and variations in atmospheric humidity and known to affect vector movements to new locations and/or act as factor in the major development of parasites (Adham et al., 2009).

In this study, older animals had a higher infection rate than young animals (22% vs. 16%). These findings support the view that calves older than one year are more susceptible to developing clinical theileriosis than younger calves; however, they are not consistent with some earlier findings from India (Singh et al., 1993), Sudan (Salih et al., 2007), and El-Mansoura province, Egypt (EL-Masry et al., 2006), which suggested greater susceptibility at ages below three or five years, based prevalence rates in the younger age groups (37.5%, 32.5%, 27.77%, or 5.88%). In another study, infection rates decreased by age (age < 1 year: 26.51%; 1 < 3 years: 9.40%; 3 < 5 years: 6.99%; age > 5 years: 4.81%; Al-Hosary et al., 2015). These reports suggest that age plays a significant role in an animal's susceptibility to infection, which could be attributable to the accumulation of infections that raise protective immunity linked to immune system maturation (Al-Hosary et al., 2015).

We found that male and female infection rates of 19.3% and 20.2%, the infection may occur in male cattle because they are typically kept in barns for fattening, which prolongs their exposure to ticks, especially when tick-control programs are not properly implemented. Infected female calves typically experience stress linked to pregnancy, parturition, and milk production. Our findings contrast with the result reported in the Cappadocia region of Turkey, where a higher infection rate was seen in female cattle (87.6%) versus males (12.4%) (Abdulla et al., 2008). That finding may be explained by specific regular management practices in this region, such as the confinement of males for an indoor feeding system with minimal grazing, which protects them from tick infestations and reduces the infection rate. On the other hand, our findings concur with a reported absence of any sex difference in infection rate in cattle (Boussaadoun et al., 2015; Selim et al., 2022).

## 5 Conclusions

The sequences for the *B. bovis small subunit ribosomal RNA* gene identified in isolates from buffalo in this study (Accession Number OP081163.1) did not show 100% identity with previously deposited sequences in GenBank (maximum identity value was 99.74% with the sequence for Accession No. MN053042.1). Similarly, the sequences for the *T. annulata small subunit ribosomal RNA* gene we identified did not show 100% identity with any previously deposited sequence in GenBank (maximum identity was 99.89%). The current study provides sequences for the *T. annulata major merozoite surface antigen* and *B. bovis* and *B. bigemina spherical body protein 4* genes identified in isolates from cattle and buffalo in southern Egypt and is the first report on these three genes in *T. annulata*, *B. bovis*, and *B. bigemina* in southern Egypt.

## CRediT authorship contribution statement

Hassan Y.A.H. Mahmoud, Tetsuya Tanaka: Conceptualization, Data curation, Formal analysis, Investigation, Funding acquisition, Project administration. Hassan Y.A.H. Mahmoud, Abdelrahman A. Rady, Tetsuya Tanaka: Resources. Hassan Y.A.H. Mahmoud, Tetsuya Tanaka: Writing - Original draft. Hassan Y.A.H. Mahmoud, Tetsuya Tanaka: Writing review & editing.

## Ethics approval

Ethics in this study were approved by the research bioethics committee at the Faculty of Veterinary Medicine, South Valley University, under approval number (VM/SVU/22(1)-02).

## Funding

This work was supported by 10.13039/501100001691JSPS KAKENHI Grant Numbers JP20KK0154 and JP22H02522, and JSPS Bilateral Program Grant Number JPJSBP120206002, JPJSBP120219936, and JPJSBP1202239937, Japan. Hassan Y.A.H. Mahmoud received financial support from the Egyptian government, 10.13039/501100007335Ministry of Higher Education and Scientific Research (Faculty of Veterinary Medicine, South Valley University, Egypt) in the form of a scholarship as a post-doctor.

## Declaration of competing interest

The authors declare that they have no known competing financial interests or personal relationships that could have appeared to influence the work reported in this paper.

## Data Availability

Data will be made available on request.
